# Dual RNA-seq of Parasite and Host Reveals Gene Expression Dynamics during Filarial Worm–Mosquito Interactions

**DOI:** 10.1371/journal.pntd.0002905

**Published:** 2014-05-22

**Authors:** Young-Jun Choi, Matthew T. Aliota, George F. Mayhew, Sara M. Erickson, Bruce M. Christensen

**Affiliations:** Department of Pathobiological Sciences, University of Wisconsin-Madison, Madison, Wisconsin, United States of America; James Cook University, Australia

## Abstract

**Background:**

Parasite biology, by its very nature, cannot be understood without integrating it with that of the host, nor can the host response be adequately explained without considering the activity of the parasite. However, due to experimental limitations, molecular studies of parasite-host systems have been predominantly one-sided investigations focusing on either of the partners involved. Here, we conducted a dual RNA-seq time course analysis of filarial worm parasite and host mosquito to better understand the parasite processes underlying development in and interaction with the host tissue, from the establishment of infection to the development of infective-stage larva.

**Methodology/Principal Findings:**

Using the *Brugia malayi*–*Aedes aegypti* system, we report parasite gene transcription dynamics, which exhibited a highly ordered developmental program consisting of a series of cyclical and state-transitioning temporal patterns. In addition, we contextualized these parasite data in relation to the concurrent dynamics of the host transcriptome. Comparative analyses using uninfected tissues and different host strains revealed the influence of parasite development on host gene transcription as well as the influence of the host environment on parasite gene transcription. We also critically evaluated the life-cycle transcriptome of *B. malayi* by comparing developmental stages in the mosquito relative to those in the mammalian host, providing insight into gene expression changes underpinning the mosquito-borne parasitic lifestyle of this heteroxenous parasite.

**Conclusions/Significance:**

The data presented herein provide the research community with information to design wet lab experiments and select candidates for future study to more fully dissect the whole set of molecular interactions of both organisms in this mosquito-filarial worm symbiotic relationship. Furthermore, characterization of the transcriptional program over the complete life cycle of the parasite, including stages within the mosquito, could help devise novel targets for control strategies.

## Author Summary

In a parasitic relationship, both host and parasite genotypes influence the parameters of their relationship. Previous studies examining host-parasite systems have examined the effects of the genotype of the host or the parasite on the relationship, but due to limitations of technology, have rarely examined interactions between the two genotypes. Here, we utilized a dual RNA sequencing (RNA-seq) approach to examine the entirety of the known transcriptomes and their interactions in the dynamic process of filarial worm development in the mosquito. In addition, the unprecedented sequencing depth achieved with this technology allowed us to compare, for the first time, parasite gene expression of larval developmental stages within the intermediate host with those life cycle stages found within the mammalian definitive host. These data provide a strong foundation for understanding how *Brugia malayi* interacts with its vector's transcriptome temporally during its complex life cycle and also simultaneously provides information on how *Aedes aegypti* responds to filarial worm infection. These data are extremely valuable for future studies of the underlying mechanisms of this mosquito-filarial worm relationship.

## Introduction

Human lymphatic filariasis (LF) is one of the most debilitating of the neglected tropical diseases, causing severe morbidity as a result of stigmatizing and disabling clinical manifestations (e.g., elephantiasis and hydrocele) [1], [2]. LF results from infection with several species of mosquito-borne filarial nematodes, including *Wuchereria bancrofti*, *Brugia malayi*, or *Brugia timori*; but *W. bancrofti* is responsible for 90% of LF infections worldwide. Mosquitoes belonging to a number of different genera, including *Culex*, *Aedes*, *Mansonia*, *Anopheles*, and *Armigeres*, can serve as competent vectors. In susceptible mosquitoes, ingested microfilariae (mf) traverse the midgut epithelium and migrate to the indirect flight muscles. Migration to the head and proboscis occurs after a series of larval molts to the infective third stage (L3), which occurs in thoracic muscle cells. The L3 is then passed to the vertebrate host when the infected mosquito takes a bloodmeal [3], [4].

The ability of certain mosquito species to ingest mf of filarial worm parasites and to support their development after ingestion is an important determinant of filarial parasite transmission [5]. Interactions between mosquitoes and filarial worms can range from an almost benign commensal relationship between organisms [3], to a fatal competition resulting in death of the host [6], or to a fatal competition resulting in death of the parasite [7]. Larval development within individual flight muscle cells therefore implies a highly intimate interaction between host and parasite, and often times the number of L3s developing from ingested mf is not constant [3], [8], [9]. For mf to successfully reach the L3 stage, host tissue must provide all of the nutritional needs of the rapidly growing nematodes, while simultaneously surviving the mechanical damage caused by nematode migration and development. As a result, infection processes accompany histological changes in host muscle fibers, ranging from depletion of glycogen granules to swelling and disintegration of nuclei or mitochondria (likely altering the metabolism of these organelles) to a complete cellular degeneration following the exit of L3s [10], [11].

It is well accepted that the study of inter-species interactions are fundamental to understanding the biology of vector-borne parasites [12]–[14], and the filarial worms that cause LF are no exception. In humans, the interactions these parasites engage in with host tissue and the immune system dictate the pathobiology of LF [15], [16]. And as mentioned, the interactions between filarial worms and their mosquito intermediate hosts determine the course of parasite development to the infective-stage. Previous efforts to understand factors that influence the developmental success of filarial worms in their mosquito vectors have led to a deeper appreciation of the complexity of the parasite-host interplay (e.g., [3], [4], [17]); however, the inability to make quantitative, molecular-level observations of the parasite and the host, simultaneously, during infection continues to limit our ability to develop a coherent understanding of the fine-grained details mediating filarial worm-mosquito interactions. Past studies have been essentially one-sided investigations focusing on either of the partners. And, most have focused on host factors while failing to take parasite activity into explicit consideration. As a consequence, filarial worm molecular processes underlying development in, and interaction with, the mosquito host tissue remain poorly described. By its very nature, parasite biology cannot be understood without integrating it with that of the host, nor can the host response be adequately explained without considering parasite activity.

Accordingly, we initiated transcriptome profiling studies to assess the whole set of molecular interactions of both organisms involved in the dynamic symbiotic processes of filarial worm development in the mosquito. Advances in sequencing-based approaches to quantitative transcriptome profiling (RNA-seq) facilitated direct, integrated analysis of mixed-species samples obtained from *in vivo* infections [18], in which host tissue samples often contained minute quantities of parasite material. With the increase in sequencing depth, RNA-seq offered improved levels of sensitivity and dynamic range of detection, without the need of predefined species-specific probes (or selective amplification of parasite RNA) and reliance on hybridization of targeted oligonucleotides (e.g., northern blotting, RT-PCR and microarrays). Here, we present a time-resolved dual RNA-seq analysis of *B. malayi*-*Aedes aegypti* interactions, which investigated the temporal organization of transcriptional events in both nematode and mosquito tissue from the establishment of infection to after infective-stage parasites had completed development in the mosquito. We report that parasite gene transcription dynamics exhibited a highly ordered developmental program consisting of a series of cyclical and state-transitioning temporal patterns, and we contextualized this parasite developmental program in relation to the concurrent dynamics of the host tissue transcriptome. Furthermore, we critically evaluated parasite gene expression changes during the heteroxenous life-cycle of *B. malayi* by comparing mRNA abundance in larval stages within the intermediate host relative to developmental stages within the mammalian definitive host in order to provide information on the transcriptomic features underpinning the mosquito-borne parasitic lifestyle.

## Methods

### Ethics statement

This study was carried out in strict accordance with recommendations set forth in the National Institutes of Health *Guide for the Care and Use of Laboratory Animals*. All animals and animal facilities were under the control of the School of Veterinary Medicine with oversight from the University of Wisconsin Research Animal Resource Center. The protocol was approved by the University of Wisconsin Animal Care and Use Committee (Approval #A3368-01).

### Mosquito strains and colony maintenance

*Ae. aegypti* used in this study were maintained at the University of Wisconsin-Madison as previously described [19]. *Ae. aegypti*, black-eyed Liverpool (LVP) strain supports the development of *B. malayi* mf to L3 [20]. The RED strain is a multiple marker strain previously used in quantitative trait loci (QTL) mapping of filarial worm susceptibility to *B. malayi* [21], [22] and does not support the development of *B. malayi* to L3 [23]. Three- to four-day-old mosquitoes were used for blood feeding and sucrose starved for 14 to 16 hours prior to this event.

### Exposure to infective blood meal

Mosquitoes were exposed to *B. malayi* (originally obtained from the University of Georgia NIH/NIAID Filariasis Research Reagent Resource Center) by feeding on ketamine/xylazine anesthetized gerbils, *Meriones unguiculatus*. The same animals were used for both biological replicates. Microfilaremias were determined, using blood from orbital punctures, immediately before each feeding and ranged from 90–204 mf per 20 µl of blood. Control mosquitoes were exposed to anesthetized, uninfected gerbils. Mosquitoes that fed to repletion were separated into 30×30×30 cm colony cages and maintained on 0.3 M sucrose in an environmental chamber at 26.5°±1°C, 75±10% relative humidity, and with a 16∶8 hour light∶dark photoperiod with a 90 minute crepuscular period at the beginning and end of each light period.

### Mosquito collection and verification of parasite infection

The sample groups were defined by the time post exposure (PE) to the infective blood meal, and thoracic tissues were collected every 24 hours from mosquitoes exposed to an infected or uninfected blood meal. Thoracic tissues were collected for four (RED) and eight (LVP) consecutive days, and also from non-blood-fed mosquitoes at the time of exposure (i.e., time-zero sample). For each time point, 30 thoraces were collected per strain per condition. Dissected tissues were immediately flash-frozen in microfuge tubes on dry ice and stored at −80°C until RNA isolation. At 8 days PE, an additional 30 mosquitoes for each strain were dissected, and the head, thorax, and abdomen were examined microscopically as described previously [4] to estimate infection intensity and prevalence (Table 1). The bootstrap confidence interval of Efron and Tibshirani was used to calculate 95% confidence intervals for mean intensity [24]. A complete set of replicate samples was collected using a distinct generation of mosquitoes blood-fed on the same infected animals in an independent exposure to take into account stochastic variations.

**Table 1:** Infection prevalence and intensity of *Brugia malayi* in *Aedes aegypti* during the generation of the present dataset.

Biological Replicate	*Aedes aegypti* strain	Prevalence (n = 30)*	Mean intensity (CI)†
1	LVP	93.3%	14.7 (12.0–17.3)
	RED	6.7%	1.0
2	LVP	97.0%	14.5 (12.4–17.3)
	RED	0%	0.0

*Parasites were observed during mosquito dissections by microscopy at 8 days after exposure to microfilaremic blood (90–204 mf per 20 µl).

† CI, Bootstrap 95% Confidence Interval for mean intensity.

### RNA isolation

Total RNA was isolated from female *Ae. aegypti* thoraces using the MasterPure RNA Purification Kit (Epicenter, Madison, WI), which included built-in Proteinase K and DNase treatments. A Labgen7b electric tissue grinder (Cole-Parmer, Vernon Hills, IL) was used for tissue homogenization, and RNA purity and concentration were determined spectrophotometrically (NanoDrop ND-1000; Thermo Scientific, Waltham, MA). For all samples, RNA integrity was visually assessed by denaturing gel electrophoresis using GelRed staining (Phenix Research Products, Candler, NC) and further confirmed by Agilent 2100 bioanalyzer (Agilent Technologies, Santa Clara, CA). Only quality intact RNA was used for RNA sequencing.

### Illumina library preparation and sequencing

Multiplex sequencing libraries were generated from 10 µg total RNA (per library) using Illumina's mRNA-seq sample prep kit and multiplexing sample preparation oligonucleotide kit (Illumina Inc., San Diego, CA) following the manufacturer's instructions. Separate libraries were constructed for each replicate sample from infected mosquitoes. Equal amounts of total RNA were pooled from replicates prior to library construction for uninfected blood-fed and non-blood-fed samples. Cluster amplification was performed on the Illumina cBOT following the manufacturer's protocol. Sequencing-by-synthesis on the Illumina Genome Analyzer IIx generated 2×50 bp paired-end reads. CASAVA v1.7 was used for de-multiplexing, and sequence quality was assessed based on %GC content, average base quality and sequence duplication levels.

### Multiplexing

**Figure 1:**
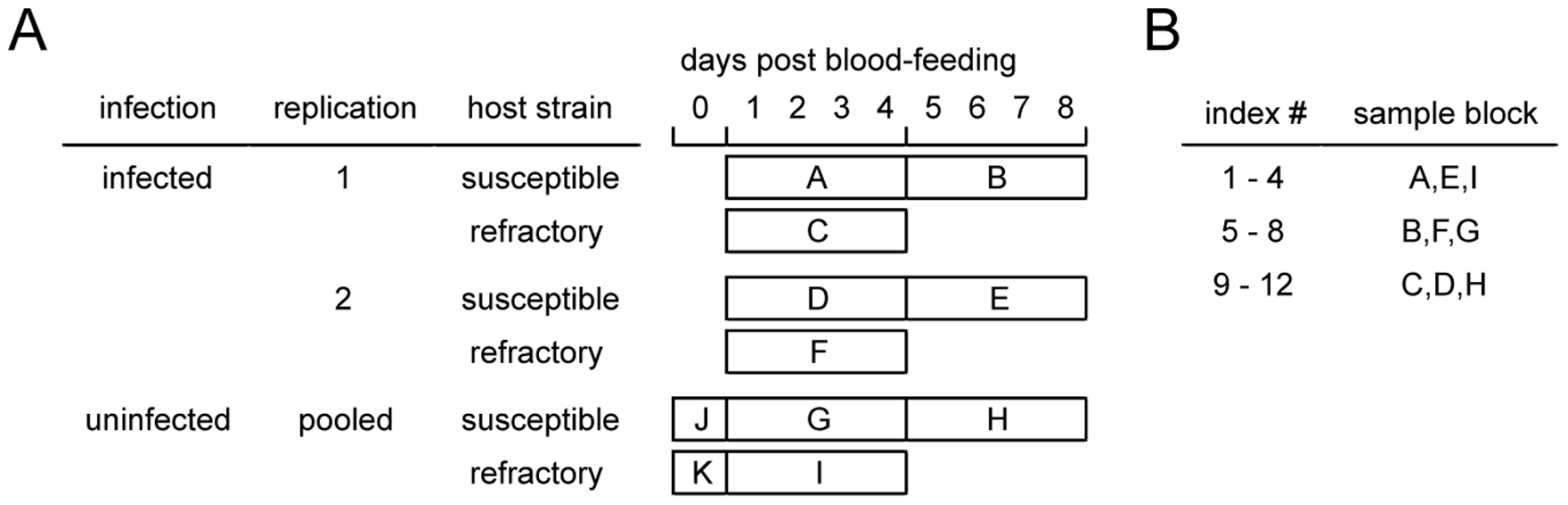
Multiplexing scheme. (**A**) Description of sequencing libraries. Except for J and K, each sample block (A through I) represents 4 distinct libraries. The following combinations of sample blocks were multiplexed without indexing conflicts: [A, B, C], [D, E, F], [G, H, I], [A, D, G], [B, E, H] and [C, F, I]. Susceptible, *Aedes aegypti* LVP; Refractory, *Aedes aegypti* RED. (**B**) Indices #1–12 were used to tag specific sample blocks. Index #1, for example, was used to tag the first library of the sample blocks A, E, and I, where each block consisted of 4 individual libraries of sequential time points. This scheme allowed multiplexing of 12 libraries from the sample blocks A, B, and C in a single lane, and also a different combination of 12 libraries from the sample blocks A, D, and G in a single lane.

To minimize the confounding effects of lane-to-lane or run-to-run technical variations, instead of sequencing each library using a single lane, libraries were sequenced over a number of lanes and runs by the use of Illumina's multiplexing capability [25]. The Illumina Genome Analyzer IIx platform allowed 12 different sequencing libraries to be multiplexed in a single lane, and 7–8 separate lanes were available during each sequencing run. Because the total number of unique libraries exceeded the maximum number of usable indices, each index was used to label multiple libraries according to the scheme outlined in Figure 1. Generation of sequence reads using these combinations of libraries (occupying 6 of the lanes during each run) contributed to a more robust dataset for longitudinal as well as cross-sectional analyses.

### Sequence alignment and transcript quantification

Sequence reads from technical replicates (i.e., reads from the same library but sequenced in different lanes and runs) were combined per library prior to mapping as they shared the same insert-size distribution. Alignment to the combined genomes of *Ae. aegypti* [VectorBase: AaegL1] and *B. malayi* [GenBank:DS236884-DS264093] was performed using TopHat v1.0.14, a splice junction mapper built upon the short read aligner Bowtie [26], [27]. The pipeline utilized exon records in the genome annotation to build a set of known splice junctions for each gene model, complementing its *de novo* junction mapping algorithm. Low quality alignments with mapping quality scores less than five were removed before downstream analyses [28], [29]. Reads aligned to exonic regions were enumerated for each gene model using the HTSeq package (v0.4.7) in Python ((www-huber.embl.de/users/anders/HTSeq). A set of paired-end reads were counted as one fragment to represent a single sampling event, and reads overlapping more than one gene model were counted as ambiguous with the mode parameter set as “union”. In addition, previously published *B. malayi* sequence reads (ArrayExpress accession number: E-MTAB-811) were aligned and quantified using an identical analysis pipeline to facilitate comparative analysis of mosquito vs. mammalian life-cycle stages [30].

### Differential transcription analysis

The RNA-seq data were prepared according to “minimum information about a high-throughput sequencing experiment” (MINSEQE) recommendations, deposited in the Gene Expression Omnibus (GEO) database, and can be accessed via the web [accession number GSE53664]. Statistical analysis of count data was performed with edgeR in Bioconductor [31]–[33]. Genes were retained for analysis if read counts were greater than 0.5 counts per million (cpm) in at least two libraries and the overall within-replicate negative binomial dispersion was lower than 0.5. Count data were normalized to account for compositional biases using trimmed mean of M (TMM) method, in addition to adjusting for differing library sizes [34]. Generalized linear models (GLM) with explanatory variables of host strain, infection status and time was fit to the count data for each mosquito gene, and the biological coefficient of variation (BCV) was inferred using the Cox-Reid profile-adjusted likelihood method [33]. For the analysis of filarial worm genes, only the host strain and time were included as factors in the GLM. Information sharing was used so that genes could take individual values for the BCV, but stabilized towards a common value [31]. To identify parasite and host genes that were differentially transcribed between any of the time points (within each time-series), we used nested interaction models, and conducted an ANOVA-like test for any differences by testing for multiple coefficients being equal to zero. Using full interaction models, we then assessed host genes that responded differently at any of the time points in the infected host relative to the uninfected host. Similarly, we tested parasite genes for differential transcription at any of the time points in the susceptible host (LVP) relative to the refractory host (RED). Adjusted p-values were reported after correction for multiple testing using the Benjamini and Hochberg method [35].

### Clustering of temporal expression profiles

All data were transformed using a log_2_ fold-change, and because log fold-change (logFC) estimates for genes with small read counts can be highly variable, we moderated logFC estimates using the ‘preFC’ function in edgeR. Thus, undefined values were avoided and poorly defined fold-changes for low counts were shrunk towards zero. We then used logFC values between sequential time points for each gene as a basis for clustering. After median-centering, K-means clustering was performed using a (uncentered) Pearson correlation as a distance metric to group and summarize expression profiles into common temporal patterns. Figure of merit (FOM) plots were utilized in determining the appropriate number of clusters to ensure informative partitioning [36]. Using goseq in Bioconductor, Gene Ontology (GO) terms (or KEGG pathways) statistically over-represented in each cluster were compiled to help interpret the biological implications of the expression patterns [37]. To assist in interpretation and visualization, we systematically summarized significant GO terms into a representative subset using an algorithm implemented in REVIGO (revigo.irb.hr) that relies on semantic similarity measures (16). GO annotation was retrieved from UniProtKB-GOA [38] and VectorBase [39].

### Multi-dimentional scaling (MDS) plot of transcriptional profiles

MDS plot (a type of unsupervised clustering) was generated in edgeR to analyze sample relationships. Distances between each pair of RNA-seq profiles corresponded to the average (root-mean-square) of the top 500 largest absolute logFC between each pair of samples.

## Results and Discussion

### Dual RNA-seq approach to analyzing parasite-host system

To study gene expression dynamics during filarial worm-mosquito interactions using RNA-seq, we collected thoracic tissue samples from *Ae. aegypti* (LVP) exposed to either a *B. malayi*-infected or uninfected bloodmeal. Thoracic tissue samples were collected every 24 hours after exposure for eight days, and also from non-blood-fed mosquitoes at the time of exposure. LVP is a genetically selected susceptible strain that supports the complete development of *B. malayi* to the infective stage [20]. In addition, a matching set of tissue samples was concurrently collected for four days using refractory *Ae. aegypti* (RED) to comparatively evaluate the transcriptome dynamics in an incompatible parasite-host association (Table 1). In this refractory mosquito, *B. malayi* mf traverse the midgut and become intracellular within the thoracic musculature, but after reaching their site for development, parasites fail to develop and die within a few days [23].

**Figure 2:**
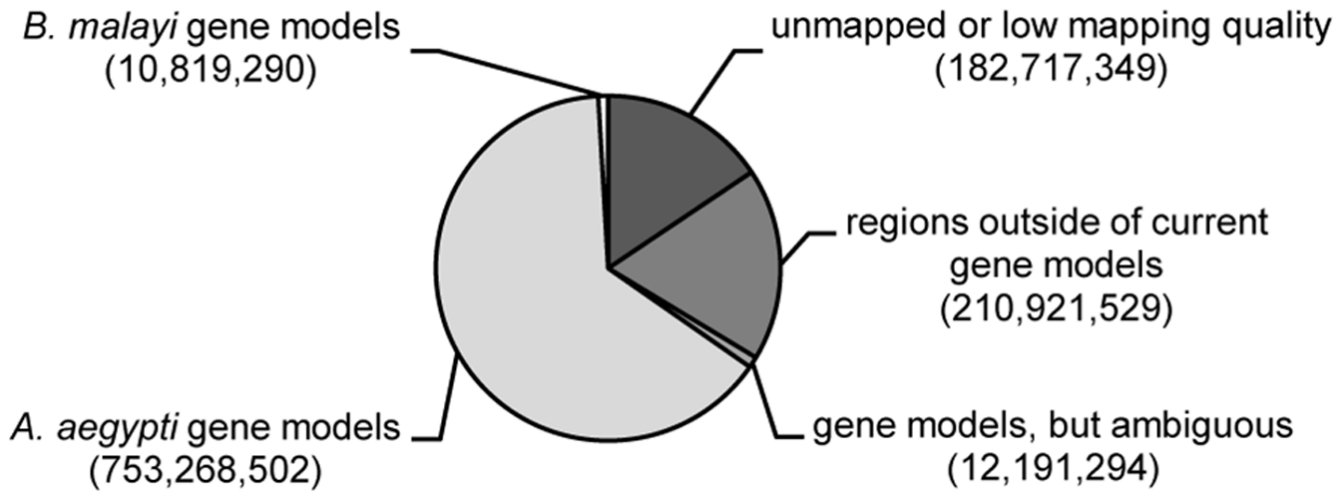
Sequencing reads aligned to the *Aedes aegypti* and *Brugia malayi* reference genomes. Overall composition of sequencing reads based on alignment to reference genomes, *Aedes aegypti* [VectorBase: AaegL1] and *Brugia malayi* [GenBank:DS236884-DS264093].

**Figure 3:**
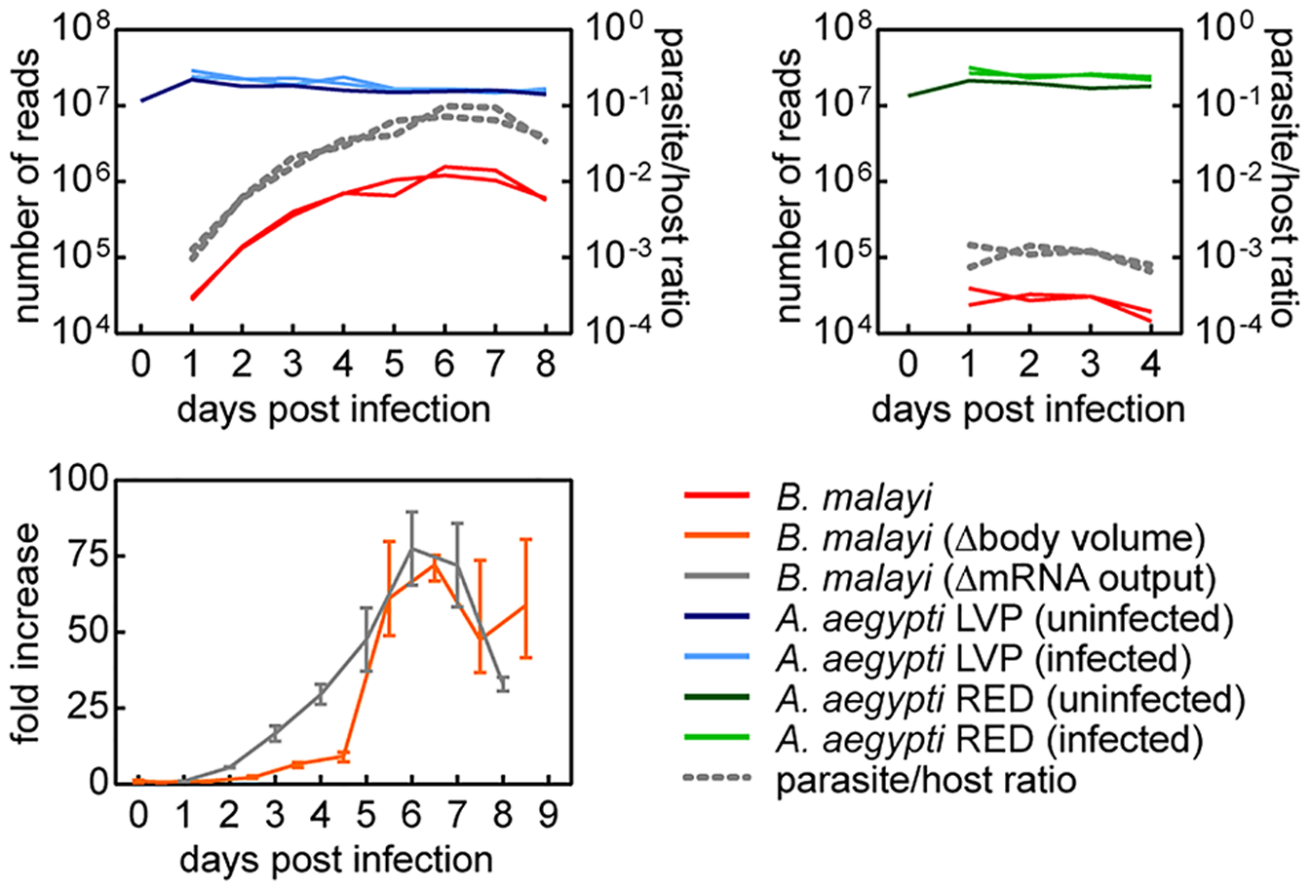
Total number of aligned paired-end reads. Upper panels represent paired-end reads that aligned unambiguously to the gene models of *Aedes aegypti* (left panel LVP, right panel RED) and *Brugia malayi*. Biological replicates were plotted as separate lines. The lower panel represents the relative changes in transcriptional output and body volume during *B. malayi* development in *Ae. aegypti* LVP. Temporal change in transcriptional output was estimated from parasite/host ratio of total read counts, assuming that the host mRNA output is constant over time. Volumetric calculations were based on measurements reported by Murthy and Sen [40] after approximating the shape of the worm to a cylinder. Error bars denote range.

Poly(A)-selected mRNA samples isolated from these tissues were subjected to Illumina sequencing (38 libraries across 48 lanes), and the resulting paired-end reads (2×50 bp) were aligned to the combined genomes of *Ae. aegypti* and *B. malayi* using a splice-junction aware aligner. Out of 1.17 billion sequenced reads, 987 million reads (84%) mapped unambiguously to the reference genomes (Figure 2). In the susceptible mosquito (LVP), the proportion of parasite reads relative to host reads increased from 0.1 to 9.0% during the course of infection, consistent with nematode growth from mf to L3 ([40] and Figure 3). Interestingly, these data suggested that during larval development leading up to the first molt (days 1–4) the rate of increase in the parasite's total mRNA output substantially exceeded that of body growth. This burst of transcriptional activity coincided with the initiation of tissue and organ differentiation following recovery from a developmental arrest during the mf stage. In the refractory mosquito (RED), the proportion of parasite reads remained at 0.1% and declined to lower levels by day 4, consistent with the observed failure of parasite development and survival (Figure 3).

**Figure 4:**
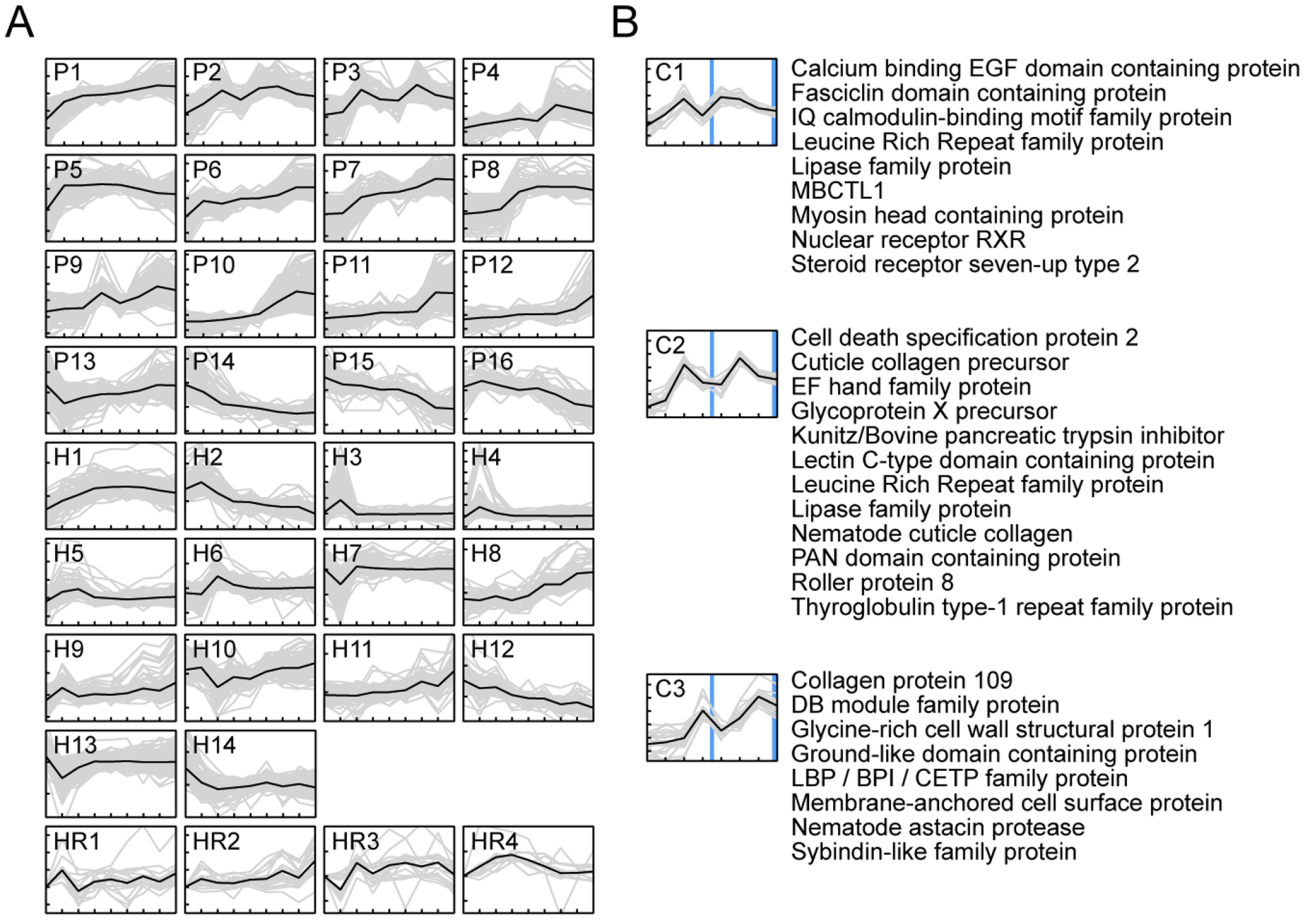
Time-course transcript abundance profiles. The vertical scale displays log_2_ fold-change in increments of 2, and the horizontal scale displays time in days. (**A**) Non-flat profiles (p<0.01 and maximum fold-difference among time points >2) were grouped into common temporal patterns using k-means clustering. Black lines represent mean expression profiles; P1–16, k-means clusters of *Brugia malayi* during development in *Aedes aegypti* LVP between day 1 and 8 post infection; H1–14, k-means clusters of *Ae. aegypti* LVP infected with *B. malayi* between day 0 and 8 post infection; and HR1–4, host response profile comparing infected vs. uninfected *A. aegypti* LVP. (**B**) *B. malayi* transcripts displaying periodic patterns with respect to molting events (blue vertical lines) during development in *Ae. aegypti* LVP. For each temporal pattern with distinct kinetics the top 20 transcripts displaying the greatest magnitude of change were plotted along with their respective gene descriptions (C1–3). Uninformative annotations (e.g., hypothetical proteins) were omitted.

**Figure 5:**
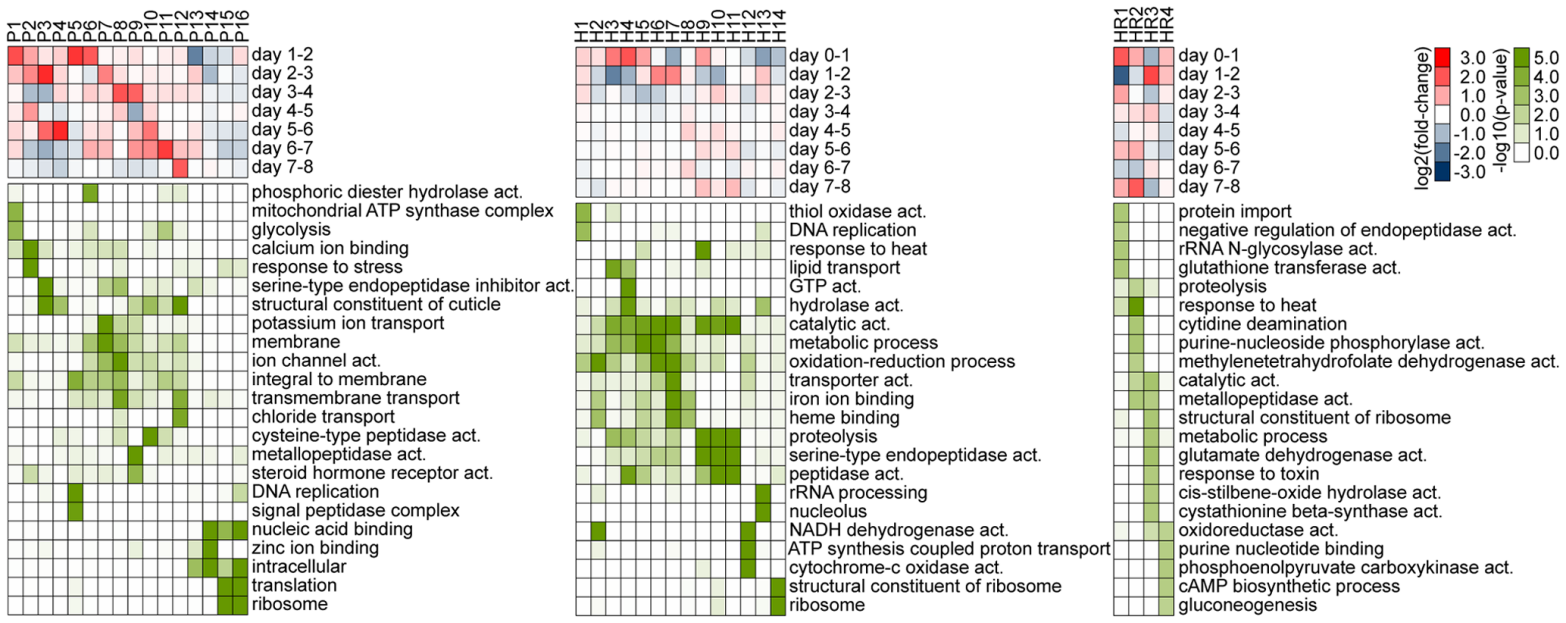
Temporal expression dynamics across clusters. Mean expression changes between sequential time points (red-blue), and over-represented Gene Ontology (GO) terms (green-white) for gene clusters shown in Figure 4A. Significant GO terms were summarized into a representative subset (see Dataset S1 for a full list of terms).

Next, in both parasite and host, we examined genome-wide temporal changes in transcript expression patterns in an effort to better understand the dynamic progression of infection processes (Figure 4 and 5). Genes displaying statistically significant differences in mRNA abundance levels across time points were identified (negative binomial generalized linear model, likelihood ratio test, p<0.01 and fold-difference >2). These “non-flat” profiles were grouped and summarized into common temporal patterns using k-means clustering. Mean expression changes between sequential time points were computed and visualized to better evaluate the temporal dynamics across clusters in reference to specific time periods. In addition, Gene Ontology (GO) terms statistically over-represented in each cluster were compiled to help interpret the biological implications of expression patterns (Figure 5).

**Figure 6:**
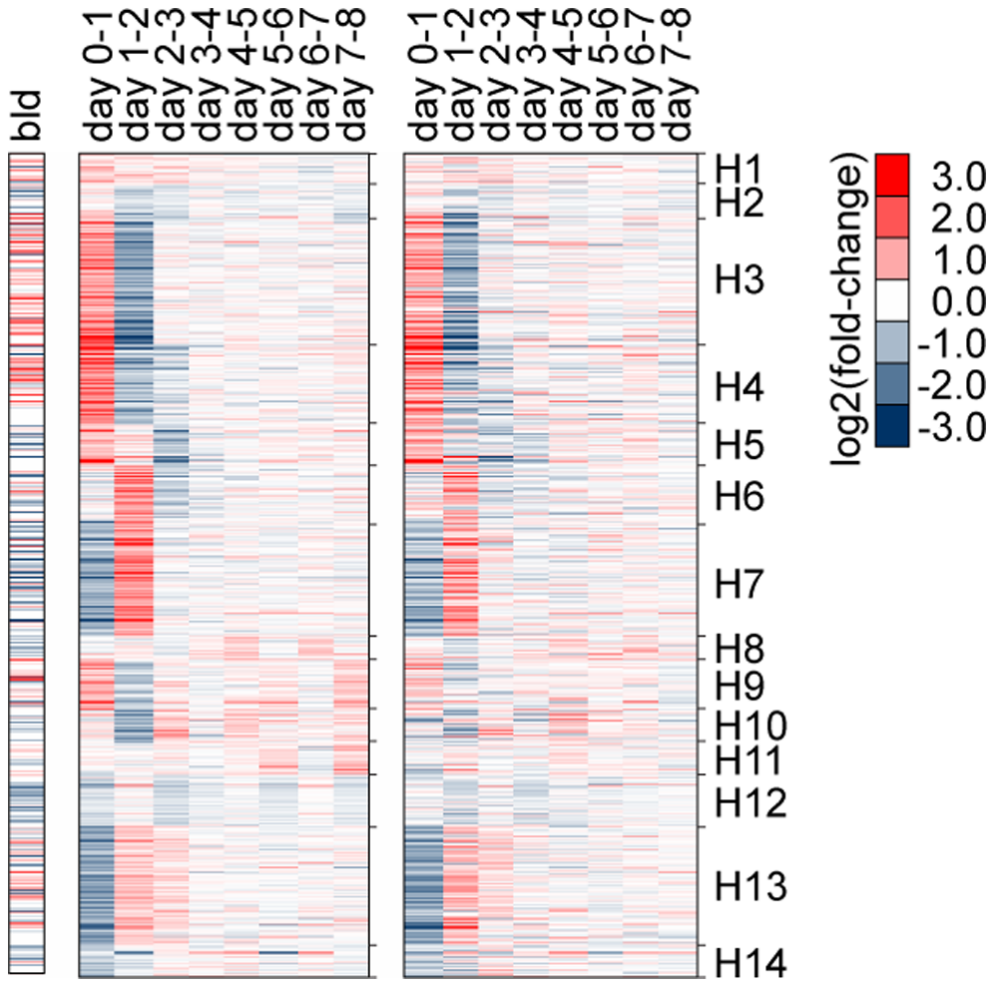
Functional composition of mosquito transcripts. Transcript abundance changes in *Aedes aegypti* gene clusters H1–14 (Figures 4 and 5) as compared to blood vs. sugar fed whole mosquitoes at five hours after bloodmeal reported in Bonizzoni et al. (bld) [43]. *Brugia malayi*-infected (middle heatmap) and uninfected *Aedes aegypti* LVP (right heatmap) thoracic tissues between day zero and eight after bloodmeal (middle and right heat maps, respectively).

An overall assessment of the transcriptional dynamics in *B. malayi* during successful development from mf to L3 indicated a progressive and complex turnover in transcriptional contents (Figure 4 and 5). Abundant transcripts at day one diminished over time, and new sets of transcripts emerged as the parasite developed. Although some expression clusters displayed patterns of gradual incremental changes, a more prevalent cluster type was marked by patterns of disproportionately larger change at specific time intervals followed by transition to a new steady level. Collectively, these state-transitioning patterns form a series of transcriptional “waves”, characteristic of developmental programs imposing order on cellular biogenesis [41]. Cyclical patterns also were evident, consistent with gene transcription associated with recurrent processes underlying nematode molting [42]. The thoracic tissue of the mosquito in which filarial worms developed also underwent extensive transcriptional changes (Figure 4 and 5). Comparisons of infected and uninfected host profiles indicated that normal physiological activities of the mosquito, most notably those related to blood-feeding responses, were accountable for a large proportion of these time-dependent expression changes ([43] and Figure 6). Concordantly, overall transcriptome dynamics were considerably higher during the first two days after blood feeding. Infection-induced gene expression changes, on the other hand, exhibited distinct temporal kinetics that appeared to reflect closely the histological phenotypes previously described in filarial worm infected *Ae. aegypti* muscle tissues (e.g., [10], [11]). In the following sections, we describe and discuss the temporal coordination of parasite and host gene expression along the developmental timeline of filarial worm-mosquito interactions.

### Gene transcription dynamics from mf to L1

After ingestion with the bloodmeal, mf traverse the midgut epithelium within two hours and begin their migration through the hemocoel to the thoracic flight muscles [19]. Myofibrils are parted to form tunnels as larvae enter host cells, within which they lie uncoiled and parallel to muscle fibers [10]. Over the next two days, mf transform into shorter, sausage form larvae (L1). This process involves rearrangement and growth of preexisting microfilarial structures, as well as extensive cuticular reorganization [44]. RNA-seq expression profiles of *B. malayi* indicated that, between days one and two, transcriptional induction was prominent among gene clusters P1, P5 and P6 (Figure 4A). GO categories associated with glycolysis, mitochondrial hydrogen-transporting ATP synthase, signal peptidase complex, DNA replication, and phosphoric diester hydrolase activity were overrepresented in these clusters. These data suggest that filarial worms respond transcriptionally to their changing metabolic and energy needs in the intracellular environment of their new host during re-initiation of larval development. Induction of genes involved in DNA replication likely is important for supporting increased cell division required for tissue differentiation. During this period, a decreasing trend was observed in clusters P13 and P14, and in the latter group, the pattern extended to day three. Genes with nucleic acid or zinc ion binding activity were highly represented in these clusters, many of which are C2H2-type zinc finger proteins that may serve as *trans* regulators of gene expression [45].

In the mosquito host, a proportion of both parasitized and non-parasitized muscle fibers show signs of structural abnormality. Within the first two days after infection, nuclear and mitochondrial enlargement occurs, and the number of affected fibers, the proportion of organelles involved, and the degree of damage progressively increases as larval development proceeds. Although the proportion is very low, severe damage and cellular degeneration, likely caused by migrating mf, also appear early in infection, but without a progression in the number of affected fibers (see [11]). The mosquito host response profile, representing transcript levels of infected animals relative to the uninfected controls, indicated that gene clusters HR1 and HR2 (Figure 4A), containing groups of stress response genes, such as small heat shock proteins with alpha-crystallin domains, displayed impulse-like patterns with acute dynamics during early infection. The distinct temporal pattern of induction among these genes, peaking at days one, six and eight, suggested a close link between the kinetics of the transcriptional response and the degree of cellular damage and mechanical disruption the host experiences as parasites penetrate into and out of muscle fiber, or actively ingest host cell contents.

Middle to late L1 development, from day three to the first molt that occurs between day four and five, is characterized by numerous mitotic divisions, lengthening of the body, and differentiation of internal structures, including a well-defined intestine and a divided-type esophagus (the anterior region muscular, the posterior glandular) that is formed around the pharyngeal thread of the mf [46]. *B. malayi* gene clusters P7 and P8 (Figure 4A), enriched with genes implicated in ion channel activity and transmembrane transport, exhibited transcriptional increase between days two and four, and their abundance levels were maintained on subsequent days. In addition, transient transcription increases were observed among clusters P2, P3 and P9 (Figure 4A), in which GO categories involved in calcium ion binding, response to stress, serpin activity, cuticle, metallopeptidase and steroid hormone receptor activity were overrepresented. Interestingly, these clusters showed a second peak during second stage larvae (L2) development, forming a cyclical pattern with respect to molting events. By filtering on the direction (increase or decrease) and magnitude of transcript abundance changes over time points, we identified genes showing strong periodic patterns where transcript levels oscillated between high levels during intermolt periods and low levels during ecdysis (Figure 4B). Given our sampling rate, we could discern three groups with distinct kinetics, reaching their maximum levels at different times (C1–3).

Genes with pulsatile transcription dynamics identified in the present study included a number of structural and regulatory components (such as cuticular collagens, cuticle-digesting proteases and nuclear receptor transcription factors) that have been predicted to participate in various aspects of the molting process [42], [47]. *B. malayi*, like all nematodes, progresses through its life stages via molts, each of which involves synthesis and secretion of a new cuticle, followed by separation and shedding of the old cuticle. From intracellular signaling to extracellular execution, molting requires a series of complex molecular reactions under tight spatiotemporal regulation at the level of the hypodermis. Also, equally critical to successful molting is a precise coordination of tissue development throughout the animal [48]. In this context, the specific ordering of gene clusters with cyclical patterns is highly intriguing and informative, shedding light on the temporal organization of molecular events underlying the periodic episodes of molting in relation to the progressive life stage transitions.

### Compensatory host response to parasite development

In addition to the initial wave of acute reactions to the invading parasites, the mosquito host's transcriptional responses to developing larvae also contained relatively moderate but sustained temporal patterns of induction, as illustrated in cluster HR4 (Figure 4A). These expression changes, extending mostly over the six to seven day period, likely reflect long-term chronic effects of filarial worm infection, part of which may represent a compensatory host response to energy and nutrient imbalances caused by intracellular parasite development. Overrepresented GO terms in this cluster included oxidoreductase activity, purine nucleotide binding, phosphoenolpyruvate carboxykinase activity, cAMP biosynthesis process, and gluconeogenesis. Because parasite-host interactions inherently involve two interconnected biological systems with a net flow of energy and nutrients, a metabolic perturbation likely is inevitable in the host [49]. It has been reported that in *Ae. aegypti*, filarial worm-infected muscle fibers show a large decrease in the amount of glycogen granules [44], and our data point to a transcriptional induction in the pathway of gluconeogenesis, by which cells synthesize glucose from metabolic precursors, such as glycogen. Data further suggest that this shift in metabolic state likely involved regulation at the level of cAMP synthesis and phosphoenolpyruvate carboxykinase activity, the latter of which controls a rate-limiting step of the pathway. Another notable feature of the infected host tissue transcriptome was the observed expression changes in glutathione transferase and glutathione peroxidase, whose main functions are to inactivate toxic products of oxygen metabolism. Induction of these antioxidative enzymes could be functioning to enhance protection from oxidative damage, or alternatively, this also may change the redox state of the host cell environment to favor parasite survival [50].

### Gene transcription dynamics from L2 to L3

**Figure 7:**
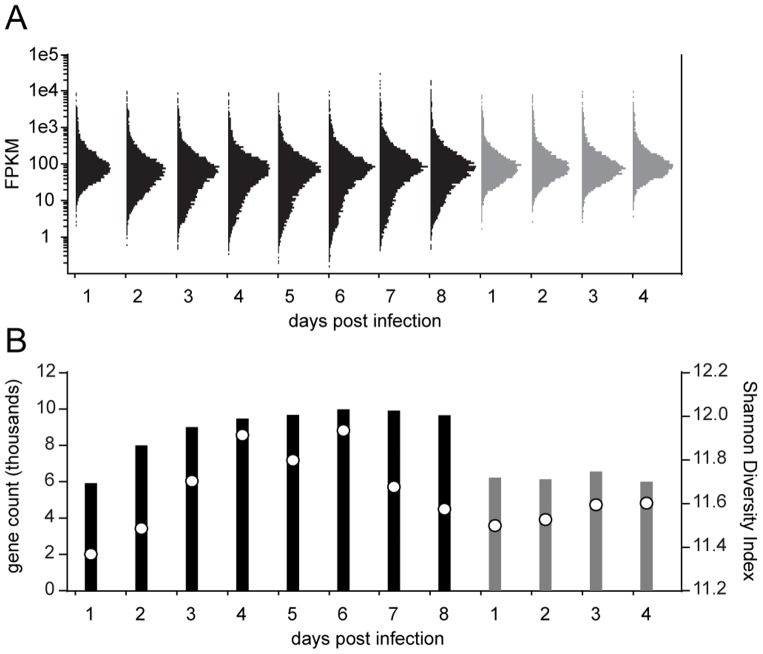
Sampling depth increased over time. (**A**) Frequency distribution of *Brugia malayi* genes over FPKM (fragments per kilobase of exon model per million fragments mapped) values. Black and grey: development in *Aedes aegypti* LVP and RED, respectively. The area of each distribution curve is proportional to the total number of detected genes for each condition (FPKM>0) (**B**). Circles represent Shannon Diversity Index values. These data show that increase in sequence sampling depth due to parasite growth (Figure 3) results in the detection of additional genes, mostly transcribed at lower levels, as evidenced by the asymmetrical broadening of shoulders in histograms.

By day five, the first molt is complete, which marks the transition between L1 and L2. At this time, L2s begin feeding through their open stoma and newly developed pharynx with a complete cuticularized lumen. Flight muscle mitochondria of the mosquito appear within the larval esophagus and midgut after six days of development, indicating active tissue ingestion [51]. During the following days, the larvae elongate and the gut undergoes further development. The rectum, however, remains closed with an anal plug, preventing egress of larval gut contents into host tissue. This could represent one of the mechanisms by which filarial worms restrict trauma to the host cell during their intracellular development [52]. The lumen of the rectum is then formed within the anal vesicle just before the molt to the third stage [46]. Between days five and six, the genital primordium is formed, the position of which differs in males and females; it is at or just behind the esophago-intestinal junction in males, and at the midesophageal level in females [53]. During the middle to late L2 stage, the body wall consists of cuticle, chords (differentiated small dorso-ventral chords and broad lateral chords) and muscle components. A slight loosening of the cuticle at the head and tail is typically observed at day seven. By day eight, most larvae have either completed the second molt or are in the process of molting. Within a day or two, L3 migrate from the thoracic muscles through the head to the labium of the proboscis, from which they exit the mosquito during blood-feeding to infect the mammalian host. During L2 development in *B. malayi*, a diverse set of genes in clusters P2, P3 and P9, with oscillatory expression patterns, exhibited their second peak of transcription (Figure 4A). Clusters P4, P10 and P11, on the other hand, showed state-transitioning patterns with transcriptional induction between days five and seven. These likely represent specific processes that were initiated during the L2 developmental period. Overrepresented GO categories in these clusters included cysteine-type peptidase activity, structural constituent of cuticle and glycolysis. Subsequently, between days seven and eight, transcript levels increased in cluster P12, in which gene sets associated with cuticle components, transmembrane transport and chloride transport were enriched. Together, these clusters encompassed a large array of functional categories, as well as genes with unknown function. Although the magnitude of change was comparatively small, clusters P15 and P16 showed a decreasing trend over the period of L2 development (Figure 4A). Enrichment of genes implicated in ribosome, translation and nucleic acid binding in these clusters suggested that genes involved in basal cellular activity were expressed in lower levels during late L2 and L3 compared to those at earlier stages (Figure 5). Overall, these data suggest a considerable change in the organism's transcript composition and a relatively uneven distribution of transcript abundances (as supported by the decrease in Shannon Diversity Index [54] between days six and eight; Figure 7) in the second stage during which high levels of cellular differentiation and tissue development occur. Nevertheless, such interpretation requires caution because our temporal data are confounded with variations in transcript detection limits, due to the differences in sequence sampling depth over time (Figure 3 and 7).

The mosquito transcriptional response during L2 development leading to L3 emergence was largely characterized by further induction of stress response genes of the heat shock protein family, many of which were responsive at the onset of infection (clusters HR1 and HR2). These may play a crucial role in the repair process of the host cell by serving as molecular chaperones, and thereby possibly suppressing or delaying necrosis during intracellular parasite development [55]. One striking observation from past histological studies is that, despite the large increase in parasite size and host tissue consumption, almost all infected cells harboring live larvae do not undergo cellular breakdown or degeneration until larval development is complete and the L3s migrate out of the flight muscle fibers [10], [11]. It is thought that some physical or chemical factor associated with the terminal phase of larval development causes significant damage above a tolerable threshold, initiating necrosis. It remains unknown whether filarial worms actively modulate host cell survival in an effort to conserve their habitat. In *Ae. aegypti* about 5–15% of all fibers eventually degenerate completely, which represents a permanent loss of contractile capacity, likely contributing to decreased flight activity and longevity [56]–[58].

### Influence of host environment on parasite gene transcription

**Figure 8:**
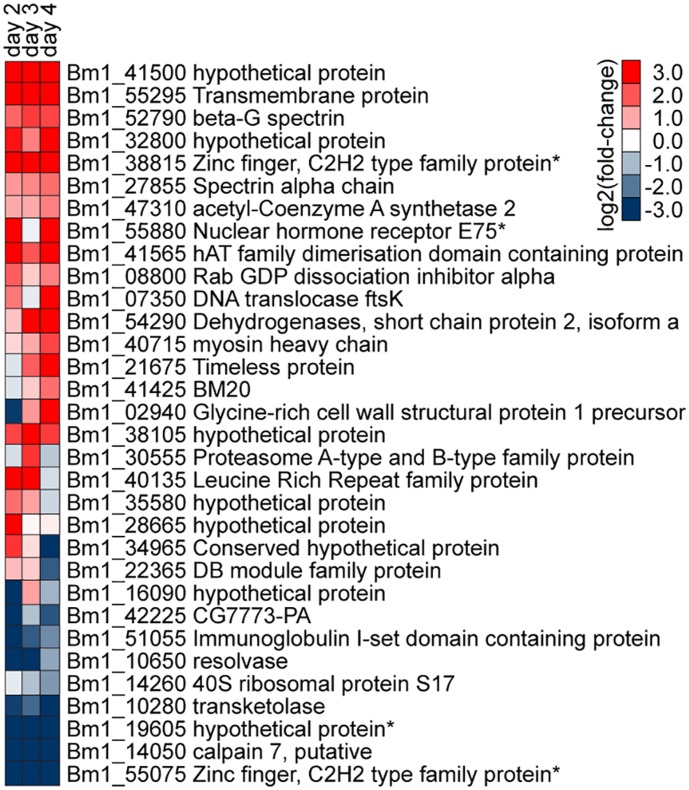
Functional composition of parasite transcripts that were different during infection of susceptible vs. refractory mosquitoes. *Brugia malayi* genes showing time dependent transcriptional changes (relative to day one) that are different during infection of *Aedes aegypti* LVP with respect to RED (i.e., log fold-change during infection of LVP – log fold-change during infection of RED). *Putative transcription factors predicted by DBD database [60].

*B. malayi* gene expression changes discussed thus far underlie successful developmental progression to the infective stage, which is dependent on the mosquito host tissue environment. Changing the genetic background of the host to an incompatible strain (RED) results in a failure of the parasite to develop and survive. Using a negative binomial generalized linear model, we assessed time dependent transcriptional changes between days one and four that were different in *B. malayi* depending on host environment, i.e., compatible (LVP) versus incompatible (RED). The most significant transcriptional alterations, as judged by statistical evidence, are presented in Figure 8 (p<0.05). Our data indicated that transcripts encoding hAT family dimerization domain containing protein, transmembrane protein, Leucine Rich Repeat family protein, dehydrogenases, and spectrins, among others, showed an increasing trend during successful parasite development. In *Caenorhabditis elegans*, a mutation in β-spectrin resulted in retarded growth and paralysis, suggesting that it is required for normal nematode development [59]. Of particular interest is the presence of putative transcriptional regulators in this list, such as nuclear hormone receptor and C2H2 type Zinc finger protein [60]. Although their specific function in *B. malayi* has not been elucidated, these could be involved in the control of key developmental pathways that are initiated during early larval development in the mosquito. Among the transcripts that showed an increasing trend in non-developing or dying worms, was a calpain gene that encodes a member of the Ca^2+^-dependent cysteine protease family. Calpain activation is an integral component of necrosis, and has been implicated in apoptotic cell death [61], which is consistent with the refractory host environment of the RED strain preventing filarial worm development, and the resultant death of the parasite (Table 1). However, because of the extensively divergent genetic backgrounds between RED and LVP, the host genetic factors that confer this lethal environment could not be reasonably inferred from the between-strain differences in the host tissue transcriptome.

### Mosquito-stage transcriptome of *B. malayi*

**Figure 9:**
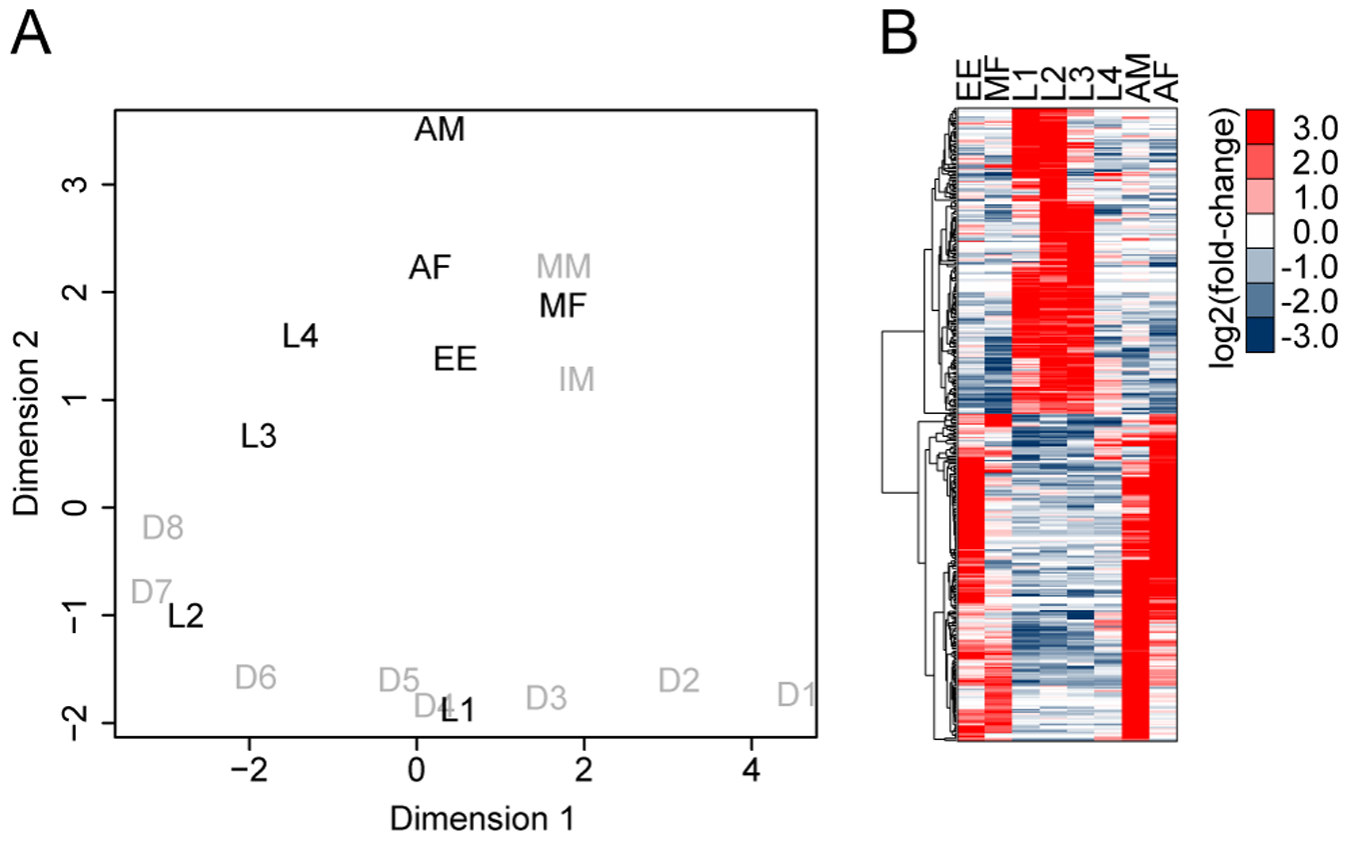
*Brugia malayi* expression profiles enriched for each life cycle stage. (**A**) Multi-dimensional scaling plot showing relatedness between transcript expression profiles of *Brugia malayi* life-cycle stages, including previously reported mammalian stages [30]. Read counts resulting from immature and mature microfilariae (IM and MM), mosquito stages between day 1 and 4 (D1–4), and between day 4 and 8 (D4–8) were combined to generate representative expression profiles enriched for microfilariae (MF), L1, and L2, respectively. AM and AF, adult male and female; EE, eggs/embryos. The resulting data set (indicated in black) were used to examine (**B**) *B. malayi* genes differentially transcribed during the mosquito stages relative to the mammalian stages (p<0.01).

To further investigate the transcriptomic features of this heteroxenous parasite in the context of host transitions, we extended our analysis to include our previously reported RNA-seq profiles (see [30]) and comparatively analyzed the mosquito stages (L1, L2, and L3) relative to the mammalian stages (egg, microfilaria, L4, adult male, and adult female). To facilitate this comparison, read count data from individual libraries were combined per stage to construct expression profiles enriched for (or representative of) each life-cycle stages (Figure 9A). Sample relations based on multi-dimensional scaling (MDS) revealed a biologically plausible pattern where expression profiles of successive stages were overall more similar to each other. From these profiles, genes differentially transcribed in the mosquito stages relative to the mammalian stages were identified (p<0.01). Of the 413 significant genes, 200 genes displayed transcriptional induction predominantly during the mosquito stages (Figure 9B). Overrepresented among these genes were GO terms, such as proteolysis, cell adhesion, oxidation-reduction process, and malate metabolic process, as well as several Molecular Function terms that appear consistent with the above-mentioned Biological Process terms (p<0.01; Dataset S1). Peptidases and peptidase inhibitors, including cathepsins, serpins and cystatins were also strongly represented, which was in agreement with previous studies giving support to their possible role in essential developmental processes or specific interactions with the host [62], [63]. Enrichment analysis using KEGG pathway further highlighted pyruvate metabolism, citrate cycle, and arachidonic acid metabolism (p<0.01; Dataset S1). A closer look at individual metabolic genes indicated a highly specific induction among the key components of anaerobic mitochondrial pathways, such as phosphoenolpyruvate carboxykinase (GenBank:Bm1_25195), malic enzyme/malate dehydrogenase (GenBank:Bm1_04060, GenBank:Bm1_08150, GenBank:Bm1_46465, GenBank:Bm1_53540), and pyruvate dehydrogenase (GenBank:Bm1_26945) [64], [65]. Adult filarial worms are homolactate fermenters that primarily produce lactate via cytosolic pathways when glucose is in excess [66]. Our data suggested that anaerobic mitochondrial metabolism involving malate dismutation is likely activated during parasite development in the mosquito, increasing the total yield of ATP per mole of glucose metabolized. A developmental regulation in energy metabolism as such could represent an important adaptive strategy for survival when glucose concentrations become limiting in the host milieu [67]. Of the 200 genes associated specifically with parasite stages in the mosquito, 51 have been either predicted to be secreted or shown to be secreted at some stage in the life-cycle (Dataset S1) [53], [68]. These molecules, including protease inhibitors, carbohydrate-binding proteins, and the abundant larval transcript (ALT) proteins, possibly constitute an important part of the parasite-host interface, some of which could potentially impact nematode survival and progression of infection.

Transcriptomic approaches have been instrumental in studying the developmental regulation of gene expression underlying filarial worm life-cycle progression [30], [69]–[72]. Advances in high-throughput nucleotide sequencing now enable us to interrogate the *in vivo* transcriptome dynamics of this metazoan parasite during its obligatory intracellular developmental phase in the mosquito host, which is critical for the maintenance of the disease cycle and transmission. Integrative analysis of the host transcriptome in a dual RNA-seq approach provides a high-resolution overview of the parasite-host system in which each partner's transcriptional state is dependent on the other partner. Studies of this nature will grow increasingly important in investigating the unique and common biological mechanisms that enable parasites to maintain their symbiotic association with the host, as well as the host strategies to counter parasitic infections, within a unified framework.

## Supporting Information

Dataset S1 A tabular representation of *Aedes aegypti* and *Brugia malayi* time course transcript abundance profiles and over-represented gene ontology (GO) terms; comparative analysis of *B. malayi* time course transcript abundance profiles during infection of compatible (LVP) versus non-compatible (RED) mosquitoes; life cycle transcript abundance profile and mosquito stage enriched expression patterns in *B. malayi*; Over-represented GO terms among the mosquito stage-enriched genes of *B. malayi*; Over-represented KEGG pathways among the mosquito stage-enriched genes of *B. malayi*; and putative secreted proteins among the mosquito stage-enriched genes of *B. malayi*. All time points and parasite stages are represented.
(XLSX)
